# Birth Outcomes in Pregnancies with Uterine Malformations: A Single-Center Retrospective Study

**DOI:** 10.3390/jcm14072379

**Published:** 2025-03-30

**Authors:** Corneliu Florin Buicu, Melinda Ildiko Mitranovici, Dan Dumitrascu Biris, Marius Craina, Elena Silvia Bernad

**Affiliations:** 1 Public Health Department, “George Emil Palade” University of Medicine, Pharmacy, Sciences and Technology, 540142 Targu Mures, Romania; florin_buicu@yahoo.com (C.F.B.); dr.dan.biris@gmail.com (D.D.B.); 2 Department of Obstetrics and Gynecology, Emergency County Hospital Hunedoara, 14 Victoriei Street, 331057 Hunedoara, Romania; 3 Department of Obstetrics and Gynecology, “Victor Babes” University of Medicine and Pharmacy, 300041 Timișoara, Romania; mariuscraina@hotmail.com (M.C.); bernad.elena@umft.ro (E.S.B.); 4 Ist Clinic of Obstetrics and Gynecology, “Pius Brinzeu” County Clinical Emergency Hospital, 300723 Timișoara, Romania; 5 Center for Laparoscopy, Laparoscopic Surgery and In Vitro Fertilization, “Victor Babes” University of Medicine and Pharmacy, 300041 Timișoara, Romania; 6 Center for Neuropsychology and Behavioral Medicine, “Victor Babes” University of Medicine and Pharmacy, 300041 Timișoara, Romania

**Keywords:** uterine malformation, Müllerian duct abnormalities, pregnancy, infertility

## Abstract

**Background and objectives:** The prevalence of uterine malformations, affecting up to 7% of the general population, is associated with high rates of pregnancy complications, such as infertility, miscarriage, preterm delivery, malpresentation, ectopic pregnancy, and other complications, with high rates of both maternal and fetal morbidity and mortality. Surgical procedures have been proposed to remediate these anomalies, with different outcomes. In this context, our study aimed to emphasize the complications encountered in our department and the pregnancy results. **Materials and Methods:** A retrospective cohort study was performed on all the women who delivered in one university-affiliated medical center between 2010 and 2017 with congenital uterine malformations. A total of 62 women were included: 26 with uterine malformations and 36 as controls. Statistical analyses were performed with the level of statistical significance set at *p* < 0.05. **Results:** Only 53.8% of the pregnancies in women with uterine malformations ended in a live birth. The cesarean section rate was 64.3% in the study group. The only successful surgical procedure performed to restore fertility was cerclage. A lower Apgar score and a higher rate of neonate admission into the intensive care unit were observed in the study group, at 11.5% compared to 0 in the control group. The most important complication encountered with statistical significance was preterm delivery. **Conclusions:** This study demonstrated that uterine congenital malformations are an independent risk factor for pregnancy complications.

## 1. Introduction

Uterine malformations have been reported to occur in up to 7% of the general population and in 3.4–8% of those who are infertile [1,2,3,4]. The prevalence of congenital uterine anomalies may be affected by variations between studies due to inaccurate diagnostic techniques [5]. Women with uterine malformations have higher rates of pregnancy complications, such as preterm delivery, miscarriage, malpresentation, cesarean section, fetal death, ectopic pregnancy, placental abruption, IUGR, and other obstetric complications with perinatal mortality [4,5,6,7,8,9,10]. The secondary outcomes include severe maternal morbidity [6,10].

Many classifications have been proposed, with the most accurate being the American Society of Reproductive Medicine’s classification [1,11]; however, the recently introduced European Society of Human reproduction and Embryology/European Society for Gynecological Endoscopy (ESHRE/ESGE) classification seems to be new, clear, and important for its clinical impact, which is also related to pregnancy outcomes [2]. The new ESHRE/ESGE classification system may increase the number of remedial surgical procedures for women previously diagnosed with arcuate uteri who are now being reclassified as having partial septate uteri [12]. In terms of female genital tract congenital anomalies, the new classification system includes five main classes: class I includes dysmorphic T-shaped and infantile uteri; class II is septate uteri, including arcuate uteri; class III includes uterus didelphys and bicornuate uteri; class IV includes unicorporeal or unicornuate uteri; and class V is aplastic uteri. Normal uteri are categorized as class 0 and unclassified cases as class 6 [2,13]. The diagnostic tools used to identify congenital uterine anomalies (CUAs), in addition to hysterosalpingography (HSG), include 3D ultrasound (3D US), magnetic resonance imaging (MRI), hysteroscopies, and laparoscopies [2]. Based on the 2013 classification by the ESHRE (European Society of Human Reproduction and Embryology) and the ESGE (European Society for Gynaecological Endoscopy), uterine anomalies are U2 [septate], U3 [bicorporeal], or U4 [hemi-uterus] [2,5].

According to most studies [4], uterine anomalies, especially bicornuate uteri, have poorer reproductive outcomes.

Urinary defects may also be present [8], and less than half have clinical symptoms [8]. The main clinical findings are amenorrhea, pelvic pain, dysmenorrhea, and sexual difficulties, with a major impact on quality of life. The diagnostic methods include a two- or three-dimensional ultrasound, MRI, hysterosalpingo-contrast sonography, X-ray hysterosalpingography, laparoscopy, and hysteroscopy [3]. According to researchers, a laparoscopy improves uterine structure detection compared to MRI alone [14,15]. The most effective diagnostic tool seems to be three-dimensional ultrasonography [2,8,16,17,18]. Abnormal Müllerian duct formation during the fetal phase of life is the cause of malformations [2]. These anatomic disorders may occur due to genetic mutations or acquired defects [8]. Genetic findings have shown that only four genes are clearly linked to Müllerian anomalies in humans. The effectiveness of treatment for these anatomic disorders remains controversial [2]. Many surgical options are available, but robust evidence of their effectiveness in improving obstetrical outcomes is lacking, as are randomized trials. There is thus a need to develop a clear study investigating these innovative surgical methods for the various anomaly categories, which would help in optimally managing these patients [1,8].

Due to their rarity, congenital uterine malformations have been relatively understudied [6]. Many cases are also difficult to detect due to a lack of awareness [5]. In this context, our study aimed to examine the complications encountered in our department and the results of pregnancies. We also explored diagnostic tools and different surgical techniques to identify their efficacy. The ultimate goal was to raise awareness of the importance of these defects among both professionals and patients in order to find the most effective methods to improve pregnancy outcomes based on the retrospective data obtained through our study.

## 2. Materials and Methods

We conducted a retrospective cohort study on all the women who delivered at one university-affiliated medical center between 2010 and 2017 with congenital uterine malformations. The inclusion criteria were as follows: Müllerian agenesia, unicornuate uterus, uterus didelphys, bicornuate uterus, septate uterus, and only in the context of infertility or pregnancy. Infertility was considered where patients were struggling to achieve a pregnancy before or after 12 months according to WHO criteria [11]. We could not include recurrent pregnancy loss simply because we did not encounter this situation in our center. Multiple pregnancies and/or those complicated by fetal anomalies were excluded. Complex malformations were also excluded. Women with a congenital uterine malformation were included in the study group (n = 26, 41.9%), and those with normal uteri were included in the reference group (n = 36, 58.1%) (total: n = 62). The maternal demographics and obstetric characteristics were recorded. The diagnostic tool used was ultrasonography; most of the time, hysteroscopy and laparoscopy are used as surgical procedures for treatment. Surgical procedures were the perfect technique to confirm the diagnosis suggested by ultrasound, and we considered it a treatment tool because surgery was used for this specific purpose. Also, combinations of techniques were not encountered in our retrospective study. The maternal and short-term neonatal outcomes were evaluated and compared between the two groups. The following outcomes were evaluated: Apgar score, birth weight, sex, and intensive care unit admission. All the patients provided signed informed consent upon admission to our department. The research protocol was approved by the ethics committee of the Clinical Studies of Emergency County Hospital Al Simionescu Hunedoara (ethical approval number: 16552/04.11.2024).

### Statistical Analysis

The distribution normality of numerical variables was assessed using the Kolmogorov–Smirnov test. Numerical data are expressed as means (standard deviation) or as medians (interquartile range) for normally and non-normally distributed data, respectively.

Differences in maternal booking characteristics and pregnancy outcomes were compared between the two subgroups of women with ANOVA or Kruskal–Wallis tests (for numerical parametric or non-parametric data); significance values were adjusted for the number of comparisons in post hoc tests. The Chi-squared test was used to compare categorical variables. The tables provide the overall *p*-values, ANOVA odds ratios, and Kruskal–Wallis or Chi-squared test results, with between-group comparisons indicated within individual cells. The level of statistical significance was set at *p* < 0.05, and all statistical tests were performed with SPSS, version 23.0.0 (SPSS Inc., Chicago, IL, USA).

## 3. Results

After adjustment for confounding factors (age, BMI, smoking, location, and school), the maternal demographics and obstetric characteristics were similar between women with uterine anomalies and those with normal uteri.

The complications associated with uterine malformations are presented in the table below, and only preterm birth was found to have increased statistical significance compared to pregnancies in women with normal uteri. The most effective diagnostic tool was the ultrasound technique (Table 1).

Only 53.8% of the pregnancies in women with uterine malformations ended with a live birth, for which the use of a cesarean section was 34.6% (significantly) higher than in the control group. However, only 26 patients with uterine malformations gave birth to a live fetus, of which 64.3% delivered through cesarean section. A low Apgar score and admission to the intensive care unit were also more frequent in the malformation group (Table 2).

Some complications were encountered—for example, breech presentation with placenta previa because of the small uterine cavity found in anomalies. Thus, we have chosen to present all these variations in Table 3.

In one case, serious bleeding occurred, associated with miscarriage. In another case, placenta previa in the case of bleeding was a serious factor in inducing preterm birth. In our study, infertility was associated with Müllerian agenesia, a bicornuate uterus, and a complete septate uterus. Miscarriages were more frequent in women with a septate, bicornuate, or unicornuate uterus compared to those with a normal uterus (19.2% compared to 8.3% in the control group). Metroplasty is not a successful intervention for a bicornuate uterus, and it was not performed in our hospital. The most successful intervention was a cerclage, which was used in six cases, leading to five live births and one miscarriage. In addition, a hysteroscopic septum resection was successful in only one case. The pregnancy outcomes were poorer in the malformation group than in the control group, especially in cases of Müllerian agenesia, a bicornuate or unicornuate uterus with a rudimentary horn, or a septate uterus. The best outcomes were obtained in those with a subseptate uterus and uterus didelphys. A breech presentation, placenta previa, and premature birth were more frequent in the malformation group than in those with a normal uterus. In one situation, an ectopic pregnancy was encountered in the rudimentary horn, followed by a hysterectomy (Table 4).

## 4. Discussion

In our study, poor pregnancy outcomes were observed in women with uterine malformations. Only 53.8% ended with a live birth, with statistically significant complications, meaning 14 patients of 26 had uterine malformations compared to 91.66% in the control group; 6 patients in the study group suffered miscarriage, which is 23.07%, compared with 9.09% in the control group. According to our study, the poorest outcomes were found for Müllerian agenesia (infertility), bicornuate uteri (infertility and miscarriage), and complete septate uteri (infertility and miscarriage with serious bleeding). According to Kong et al.’s study (2024), Müllerian unification and canalization defects could be associated with lower live birth rates and higher early miscarriage rates [19]

Metroplasty does not increase the rate of pregnancy, similarly to the results of Pedro (1993) [4]; however, in Wang et al.’s study (2019), the infertility rate was significantly higher after a metroplasty in patients with a complete uterine septum [20]. Other studies, such as Ganti et al.’s (2024), suggested that hysteroscopic metroplasty can reduce pregnancy-related complications [21]. In our study, a small uterine cavity was obtained after the metroplasty.

According to Al Bdairi et al.’s study (2024), a higher prevalence of congenital uterine anomalies was found among patients with an ectopic pregnancy [13]. In our study, an ectopic pregnancy in the rudimentary uterine horn was observed, followed by a hysterectomy. In addition, compared to women without anomalies, women with congenital uterine abnormalities had a significantly higher risk of developing infertility, an ectopic pregnancy, or abortions; this is similar to other findings in the literature [13]. According to Pedro [4], poorer pregnancy outcomes were found in the bicornuate and arcuate uterus groups; the same was found in our study and that of Kong (2024). Here, we should insist on the underdiagnosis of subseptate uteri, which can be confused with arcuate uteri, a form of subseptate uteri according to ESHRE classification [22]. In our study, we achieved a successful diagnosis using ultrasound, also being confirmed after surgical procedures used as treatment. According to the ASRM classification, an arcuate uterus is a variation of a normal uterus [23]. In this regard, a careful investigation of these cases is recommended.

Cerclage may be employed in the majority of cases, with good results [4]. We used cerclage successfully in six cases—in unicornuate, septate, subseptate, and didelphys uteri—with five live births, cervical incompetence not being associated in our cases. Cerclage was placed at the gestational age of 14 weeks of gestation. According to other studies, women with unicornuate uteri have the poorest reproductive outcomes; arcuate uteri are not associated with pregnancy complications. A septate uterus leads to an increased miscarriage rate [24]. According to our study, the poorest outcomes were found for Müllerian agenesia, bicornuate uteri, and complete septate uteri. Preterm birth was observed in uteri didelphys, subseptate uteri, and unicornuate uteri, alone or associated with other complications such as placenta previa with bleeding or breech presentation, probably due to the small uterine cavity. Hysteroscopic resection was followed by infertility or miscarriages in our study. Unicornuate and didelphys uteri are associated with poor reproductive performances, first- and second-trimester miscarriages, and preterm birth; however, according to De Angelis (2015), successful pregnancy is possible [25]. Septate, “T-shaped”, and bicornuate uteri are associated with the worst reproductive outcomes [25].

Shokeir (2011) performed hysteroscopic septoplasty in women with unexplained infertility [26]. Afterwards, nearly 80% of the women conceived spontaneously [26]. Metroplasty did not lead to good results in our study, but, as mentioned, this procedure was not performed in our clinic. In addition, Sánchez-Santiuste et al. (2020) performed hysteroscopic office metroplasties (HOME-DU) in infertile patients and those with recurrent pregnancy loss, achieving a high live birth rate [27]. According to other studies, surgical treatment for CUAs is not recommended because of the high risk compared to the potential benefits [28].

Different subtypes of congenital uterine malformations are associated with different obstetric risks. According to our study, the highest risk of preterm birth was encountered in uterine didelphys (100%), followed by subseptate and arcuate uteri. An arcuate uterus was an independent risk factor for a poor pregnancy outcome in studies by Connolly et al. (2021) [29] and Kadour Peero et al. (2022) [27]. Didelphys uteri had a higher risk of preterm delivery [30].

A study regarding pregnancy outcomes in women with uterus didelphys or a bicornuate or arcuate uterus showed relatively good outcomes; however, preterm birth, cesarean sections, and IUGR were observed in women with uterus didelphys [31].

While the most important diagnostic tool in our study was ultrasound, uterine malformations remained underdiagnosed; for example, confusion between arcuate and subseptate uteri was settled, and ectopic pregnancy in the woman with a unicornuate uterus and a rudimentary horn was diagnosed through laparotomy. The same observation was made by Li et al. (2019), who noted that rupture can occur in the first trimester of rudimentary horn pregnancies [32]. If a pregnancy in the rudimentary horn is diagnosed before rupture, the excision of the pregnant horn is recommended in order to save the uterus [32,33,34,35]. Other researchers have also found obstructed horns to be associated with abdominal pain or dysmenorrhea without pregnancy; the removal of the obstructed horn may be required in such cases, using laparoscopy with morcellation or laparotomy [36]. Improvements in diagnostic methods are mandatory.

While the ESHRE-ESGE guidelines for the cut-off value overestimate the prevalence of septate uteri, those of the ASRM underestimate it. Congenital Uterine Malformation by Experts (CUME) recommends considering an indentation depth  ≥  10 mm as septate [22]. In addition, to distinguish between T-shaped and normal/arcuate uteri, Congenital Uterine Malformation by Experts (CUME) uses a new methodology [37]. This means that mistakes can easily be made when investigating women with these conditions, which can lead to pregnancy mismanagement and poor obstetrical outcomes. In everyday practice, the CUME definitions could help in diagnosing uterine morphology and enriching uterine malformation classification systems. According to Wang et al., infertility cannot be caused by a non-obstructive uterine malformation, but the volume of the uterine cavity may be associated with first-trimester miscarriage. Transvaginal three-dimensional ultrasonography appears to be the best option for diagnosis [38].

In other recent studies, arcuate uteri are still considered uterine malformations, even though their impact on pregnancy is not aggressive [39]. An arcuate uterus is the mildest form of a uterine anomaly [5]. In their recent study (2024), Yoshihara et al. considered how an arcuate uterus could be an independent risk factor for different pregnancy complications such as preterm delivery, IUGR, and abnormal placental cord insertion [40].

In addition, Cahen-Peretz et al. (2019) compared short-term neonatal morbidity and mortality in the pregnancies of women with and without uterine anomalies [41]. In our study, a low Apgar score and intensive care unit admissions of neonates delivered by women with uterine malformation pregnancies were significantly more common than in the control group. In a case report by Abe et al., the authors reported severe cerebral palsy in a neonate after a pregnancy associated with a bicornuate uterus [42]. Müllerian anomalies were considered an independent risk factor for pregnancy complications such as pathological placental abruption, a cesarean section, intrauterine growth restriction, pre-eclampsia, and perinatal mortality due to preterm delivery or a small-for-gestational-age neonate [41,43]. Abnormal placental cord insertion, which is associated with poor pregnancy outcomes, was more frequent in pregnancies complicated by CUA [40,44].

These congenital malformations have not yet been accurately defined. Most studies have reported an increased prevalence of congenital uterine anomalies in patients experiencing miscarriage or infertility [5]. Two of our patients achieved pregnancy through ART techniques, one with a septate uterus resulted in a miscarriage, and one with a subseptate uterus resulted in a live birth. The female reproductive tract develops within the first six weeks of an embryo’s life, from two Müllerian ducts, through migration, differentiation, fusion, and absorption. Knowing the anatomy of the uterus helps in identifying its possible congenital malformations. Any interruptions to this complex process can cause anomalies [5,45]. Successful embryo implantation and placenta formation depend on a proper endometrial cavity; any defects can lead to infertility or other pregnancy complications [5].

The absence of a universally accepted classification system interferes with the proper management of these conditions. Patients with CUA should be incorporated into risk-stratification for pregnancy complications in order to find proper prevention strategies [46].

In a large study conducted by Mandelbaum et al. in 2024, the majority of patients with CUA experience viable deliveries, but a proper classification is needed because different obstetric outcomes are observed for each anomaly subtype. These kinds of studies may help inform patients with CUA considering pregnancy [39].

CUA can also be easily misdiagnosed. For example, during one year at a tertiary center, three anomalies did not receive prior diagnosis and treatment, and were only discovered during cesarean section [47]. Our study emphasized that the most important complication encountered after the proper management of pregnancies in women with CUA was premature delivery, but different types of malformation represent risk factors for different pregnancy complications, similar to data in the literature [30,39]. However, prematurity can also be associated with other conditions such as breech presentation, abruptio placentae, placenta previa, or intrauterine growth restriction [43,44]. Abhinaya et al. conducted a study (2024) in a tertiary center, finding that CUA requires proper counseling in the antenatal period and proper monitoring during labor [48,49]. Cervical cerclage and elective cesarean section could help in properly managing pregnancies with CUA [49]. Furthermore, different diagnostic modalities are necessary for precise diagnosis in such situations [49,50].

The strength of our study lies in its evaluation of uterine malformations in our service, with the possibility of appropriate surgical interventions in the case of pregnancy complications. A retrospective study is quick and inexpensive, also identifying issues that help us design a future prospective study. The number of cases encountered corresponds to the data in the literature. We were also able to evaluate the evolution of pregnancies and infertility and draw conclusions. Women with uterine malformations should be informed regarding the impact of anomalies on the chances of fertility and pregnancy-related complications. However, we found a lack of awareness in both patients and gynecologists. One limitation of our study was that the diagnostic tool used most of the time during pregnancy was ultrasound. Another is that it is an observational retrospective study conducted at a single center, thus limiting the conclusions. To attempt to evaluate the efficacy of diagnostic tools or surgical interventions is associated with a lack of validity. Also, there is no proper comparison with a control group. Different events, other than the intervention, can influence the outcomes; also, the selection mode of the instruments in the future can influence the results, which clearly limits the interpretation.

The small sample size was dictated by findings from the literature, although anomalies are rare in the general population according to ASRM [23]. For example, in a tertiary center retrospective analysis, only 15 patients with Müllerian anomalies were treated in the past 6 years [36]. This represents a significant challenge to women and their clinicians in pregnancy management.

Our study has clinical relevance because it clearly emphasizes the importance of the ultrasound diagnosis of malformations, using clinical guidelines and recommendations, and raising awareness among patients regarding the possible complications. Also, even if hysteroscopic septoplasties were followed by infertility and miscarriage in our study, we encourage the patients to continue to try to obtain pregnancies. For example, new tools such as monopolar/bipolar resectoscopes enhance clinical outcomes [37,51]. Monitoring techniques for subtypes may enhance outcomes, which would greatly increase their clinical significance. Also, to incorporate multidisciplinary approaches with a close coordination between obstetricians, gynecologists, and urologists—particularly when dealing with complicated congenital abnormalities—is the most appropriate management of these pathologies. Even though the pregnancy success rate for patients with uterine malformations has been low, good practice using a proper classification of malformations and awareness of these pathologies increases the chance of future pregnancies by finding new treatment methods. In our retrospective study, we found that a good assessment of the situation and the accuracy of the diagnosis led to the achievement of pregnancy by adapting the therapy to the situation.

## 5. Conclusions

In our study, we demonstrated that uterine congenital malformations are an independent risk factor for pregnancy complications, with only 53.8% of these pregnancies resulting in live births. A statistically significantly higher rate of premature delivery was observed, as were lower Apgar scores and neonate admissions to the intensive care unit; however, no statistical significance was encountered. A wide variation exists between studies due to the inaccuracy of diagnostic tests and non-standardized classification systems. Accurate information on the limitations and efficacy of various diagnostic tools, classification systems, and different surgical options, followed by different obstetric outcomes, will help in finding optimal management practices for patients with uterine malformations. Further studies are needed in this regard.

## Figures and Tables

**Table 1:** Maternal outcomes and interventions.

Variable	Group 1 = Control Group (n = 36)	Group 2 = Analyzed Group (n = 26)	95% CI of Difference	*p*-Value	Odds Ratios
Age	24.0–31.0	23.7–30.3	−3.16 to 2.92	*p* = 0.94	-
Living in the city, n (%)	23 (63.9)	15 (57.7)	0.35 to 2.98	*p* = 0.63	1.06
Education					
Elementary school, n (%)	4 (11.1)	3 (11.5)	0.24 to 4.09	*p* = 0.46	0.95
High school, n (%)	22 (61.1)	12 (46.2)	0.63 to 4.88	*p* = 0.30	1.8
University, n (%)	10 (27.8)	11 (42.3)	0.17 to 1.47	*p* = 0.28	0.52
BMI > 25, n (%)	10 (27.8)	6 (23.1)	0.39 to 4.47	*p* = 0.77	1.3
Smoking, n (%)	20 (55.6)	11 (42.3)	0.59 to 4.55	*p* = 0.44	1.7
Genital malformations					
Bicornuate uterus	-	2 (7.7)	-	-	-
Müllerian agenesia	-	1 (3.8)	-	-	-
Septate uterus	-	5 (19.2)	-	-	-
Subseptate uterus	-	11 (42.3)	-	-	-
Unicornuate uterus	-	5 (19.2)	-	-	-
Uterus didelphys	-	2 (7.7)	-	-	-
Normal uterus	36 (100)	0		-	-
Diagnosis					
Ultrasound	36 (100)	9 (34.6)	-	-	-
Surgery	-	5 (19.2)	-	-	-
Hysteroscopy	-	8 (30.8)	-	-	-
Hysterosalpingography	-	4 (15.4)	-	-	-
Pregnancy complications					
Cesarean section < 1 year	2 (5.6)	0	-	*p* = 0.5	-
Pre-eclampsia	2 (5.6)	0 (0)	-	*p* = 0.5	-
Miscarriage	3 (8.3)	6 (23.1)	0.07 to 1.1	*p* = 0.1	0.3
Preterm birth < 37 weeks	1 (2.8)	7 (26.9)	0.006 to 0.007	***p* < 0.05**	0.07
Breech presentation	1 (2.8)	4 (15.4)	0.01 to 1.01	*p* = 0.15	0.15
Fetal growth restriction	1 (2.8)	0 (0)	-	*p* = 0.5	-

Legend: BMI = body mass index; the bold denotes statistical significance.

**Table 2:** Fetal outcomes and type of delivery.

Variable	Group 1 = Control Group (N = 36)	Group 2 = Analyzed Group (N = 26)	95% CI of Difference	*p*-Value	Odds Ratios
Birthweight	2790–3500	1800–2765	−1339 to −349.3	*p* = 0.77	-
Gender					
Male	9 (25)	6 (23)	0.38 to 4.32	*p* = 0.9	1.2
Female	24 (66.6)	8 (30.8)	1.24 to 10.9	***p* < 0.01**	4
Apgar score	6–9	9–10	−2.69 to 0.64	*p* = 0.66	-
Admission ITU	0 (0)	3 (11.5)	-	-	-
Vaginal delivery	22 (61.1)	5 (19.2)	2.14 to 19.26	***p* < 0.01**	6.6
Cesarean section	11 (30.6)	9 (34.6)	0.24 to 2.27	*p* = 0.58	0.7

Legend: BMI = body mass index; the bold denotes statistical significance.

**Table 3:** Complications associated with uterine congenital malformations.

	Complications	Groups		Total
		Control (Normal) No (%)	Study (Malformations) No (%)	
Pregnancy complication	**-**	0 (0.0)	2 (7.7)	2 (3.2)
	Breech presentation	1 (2.8)	1 (3.8)	2 (3.2)
	Breech presentation/placenta previa	0 (0.0)	1 (3.8)	1 (1.6)
	Breech presentation/preterm birth	0 (0.0)	1 (3.8)	1 (1.6)
	Cesarean section under 1 year	2 (5.6)	0 (0.0)	2 (3.2)
	DPPNI	1 (2.8)	0 (0.0)	1 (1.6)
	Pelvic dystocia	2 (5.6)	0 (0.0)	2 (3.2)
	Ectopic pregnancy in rudimentary horn	0 (0.0)	1 (3.8)	1 (1.6)
	Infertility	0 (0.0)	3 (11.5)	3 (4.8)
	IUGR	1 (2.8)	0 (0.0)	1 (1.6)
	Miscarriage	3 (8.3)	5 (19.2)	8 (12.9)
	Miscarriage with serious bleeding	0 (0.0)	1 (3.8)	1 (1.6)
	None	21 (58.3)	3 (11.5)	24 (38.7)
	Oligoamnios	1 (2.8)	0 (0.0)	1 (1.6)
	Placenta previa	1 (2.8)	1 (3.8)	2 (3.2)
	Pre-eclampsia	2 (5.6)	0 (0.0)	2 (3.2)
	Preterm birth	1 (2.8)	4 (15.4)	5 (8.1)
	Preterm birth/breech presentation	0 (0.0)	1 (3.8)	1 (1.6)
	Preterm birth/placenta previa	0 (0.0)	2 (7.7)	2 (3.2)
Total		36	26	62

Note: DPPNI—premature detachment from the normally inserted placenta; IUGR—intrauterine growth restriction.

**Table 4:** Interventions used in those with malformed vs. normal uteri.

		Groups		Total
		Control (Normal) No (%)	Study (Malformations) No (%)	
Intervention	Cerclage	0 (0.0)	5 (19.2)	5 (8.1)
	Hysterectomy	0 (0.0)	2 (7.6)	2 (2.8)
	Hysteroscopic resection	0 (0.0)	2 (7.7)	2 (3.2)
	Hysteroscopic resection/cerclage	0 (0.0)	1 (3.8)	1 (1.6)
	Metroplasty	0 (0.0)	1 (3.8)	1 (1.6)
	No	36 (100)	14 (53.8)	50 (80.6)
Total		36	26	62

## Data Availability

Raw data are available in the Al Simionescu County Hospital registers.

## References

[1] Bhagavath B., Greiner E., Griffiths K.M., Winter T., Alur-Gupta S., Richardson C., Lindheim S.R. (2017). Uterine malformations: An update of diagnosis, management, and outcomes. Obstet. Gynecol. Surv..

[2] Ludwin A., Ludwin I., Neto M.A.C., Nastri C.O., Bhagavath B., Lindheim S.R., Martins W.P. (2019). Septate uterus according to ESHRE/ESGE, ASRM and CUME definitions: Association with infertility and miscarriage, cost and warnings for women and healthcare systems. Ultrasound Obstet. Gynecol..

[3] Passos I.d.M.P.e., Britto R.L. (2020). Diagnosis and treatment of müllerian malformations. Taiwan. J. Obstet. Gynecol..

[4] Acién P. (1993). Reproductive performance of women with uterine malformations. Hum. Reprod..

[5] Kim M.-A., Kim H.S., Kim Y.-H. (2021). Reproductive, obstetric and neonatal outcomes in women with congenital uterine anomalies: A systematic review and meta-analysis. J. Clin. Med..

[6] Mandelbaum R.S., Anderson Z.S., Masjedi A.D., Violette C.J., McGough A.M., Doody K.A., Guner J.Z., Quinn M.M., Paulson R.J., Ouzounian J.G. (2024). Obstetric outcomes of women with congenital uterine anomalies in the United States. Am. J. Obstet. Gynecol. MFM.

[7] Naeh A., Sigal E., Barda S., Hallak M., Gabbay-Benziv R. (2022). The association between congenital uterine anomalies and perinatal outcomes–does type of defect matters?. J. Matern. Fetal Neonatal Med..

[8] Gliozheni O., Gliozheni E. (2021). Congenital uterine anomalies: Impact on perinatal outcomes. Orion.

[9] Solanki K., Kochar S., Poonia L. (2020). Case series on obstetrical outcomes in patient with uterine malformations. Int. J. Reprod. Contracept. Obstet. Gynecol..

[10] Bulletti C., Simon C. (2019). Bioengineered uterus: A path toward ectogenesis. Fertil. Steril..

[11] Goyal L.D., Dhaliwal B., Singh P., Ganjoo S., Goyal V. (2020). Management of mullerian development anomalies: 9 years’ experience of a tertiary care center. Gynecol. Minim. Invasive Ther..

[12] Knez J., Saridogan E., Bosch T.V.D., Mavrelos D., Ambler G., Jurkovic D. (2018). ESHRE/ESGE female genital tract anomalies classification system—The potential impact of discarding arcuate uterus on clinical practice. Hum. Reprod..

[13] Al-Bdairi A.A.H., Al-Hindy H.A.-A.M., Rahmatullah W.S., Alshukri W.S.M. (2024). Impact of Congenital Uterine Anomalies on Ectopic Pregnancy: A Cross-Sectional Observational Study of 510 Cases. Med. J. Babylon.

[14] Economy K.E., Barnewolt C., Laufer M.R. (2002). A comparison of MRI and laparoscopy in detecting pelvic structures in cases of vaginal agenesis. J. Pediatr. Adolesc. Gynecol..

[15] Vallerie A.M., Breech L.L. (2010). Update in Müllerian anomalies: Diagnosis, management, and outcomes. Curr. Opin. Obstet. Gynecol..

[16] Wang L., Chen X.-J., Liang J.-H., Zhang Z.-K., Cao T.-S., Zhang L. (2022). Preliminary application of three-dimensional printing in congenital uterine anomalies based on three-dimensional transvaginal ultrasonographic data. BMC Women’s Health.

[17] Berger A., Batzer F., Lev-Toaff A., Berry-Roberts C. (2014). Diagnostic imaging modalities for Müllerian anomalies: The case for a new gold standard. J. Minim. Invasive Gynecol..

[18] Montenegro C.A.B., Leite S.P., Mathias M.L., Rezende-Filho J. (2000). F69Three-dimensional ultrasound in the diagnosis of uterine malformations. Ultrasound Obstet. Gynecol..

[19] Kang J., Qiao J. (2024). Impact of congenital uterine anomalies on reproductive outcomes of IVF/ICSI-embryo transfer: A retrospective study. Eur. J. Med. Res..

[20] Wang X., Hou H., Yu Q. (2019). Fertility and pregnancy outcomes following hysteroscopic metroplasty of different sized uterine septa: A retrospective cohort study protocol. Medicine.

[21] Ganti S., Arogyaswamy P., Srinivasan J., Archunan P.A., Parimala A., Srinivasan J., Aarthy P. (2024). Maternofetal Outcomes in Women with Congenital Uterine Anomalies. Cureus.

[22] Ludwin A., Martins W.P., Nastri C.O., Ludwin I., Neto M.A.C., Leitão V.M., Acién M., Alcazar J.L., Benacerraf B., Condous G. (2018). Congenital Uterine Malformation by Experts (CUME): Better criteria for distinguishing between normal/arcuate and septate uterus?. Ultrasound Obstet. Gynecol..

[23] Ludwin A., Tudorache S., Martins W.P. (2022). ASRM Müllerian Anomalies Classification 2021: A critical review. Ultrasound Obstet. Gynecol..

[24] Lin P.C. (2004). Reproductive outcomes in women with uterine anomalies. J. Women’s Health.

[25] De Angelis C., Caserta D. (2015). Pregnancy outcome in women with uterine anomalies. Female Genital Tract Congenital Malformations: Classification, Diagnosis and Management.

[26] Shokeir T., Abdelshaheed M., El-Shafie M., Sherif L., Badawy A. (2011). Determinants of fertility and reproductive success after hysteroscopic septoplasty for women with unexplained primary infertility: A prospective analysis of 88 cases. Eur. J. Obstet. Gynecol. Reprod. Biol..

[27] Sánchez-Santiuste M., Ríos M., Calles L., Cuesta R.d.l., Engels V., Pereira A., Pérez-Medina T. (2020). Dysmorphic uteri: Obstetric results after hysteroscopic office metroplasty in infertile and recurrent pregnancy loss patients. a prospective observational study. J. Clin. Med..

[28] Akhtar M.A., Saravelos S.H., Li T.C., Jayaprakasan K., Royal College of Obstetricians and Gynaecologists (2025). Reproductive Implications and Management of Congenital Uterine Anomalies (2024 Second Edition) Scientific Impact Paper No. 62. BJOG Int. J. Obstet. Gynaecol..

[29] Connolly C.T., Hill M.B., Klahr R.A., Zafman K.B., Rebarber A., Fox N.S. (2021). Arcuate uterus as an independent risk factor for adverse pregnancy outcomes. Am. J. Perinatol..

[30] Kadour Peero E., Badeghiesh A., Baghlaf H., Dahan M.H. (2022). What type of uterine anomalies had an additional effect on pregnancy outcomes, compared to other uterine anomalies? An evaluation of a large population database. J. Matern. Fetal Neonatal Med..

[31] Fedele F., Bulfoni A., Parazzini F., Levi-Setti P.E., Busnelli A. (2024). Assisted reproductive technology outcomes in women with congenital uterine anomalies: A systematic review. Arch. Gynecol. Obstet..

[32] Li X., Peng P., Liu X., Chen W., Liu J., Yang J., Bian X. (2019). The pregnancy outcomes of patients with rudimentary uterine horn: A 30-year experience. PLoS ONE.

[33] Tufail A., Hashmi H.A. (2007). Uterine Horn. J. Coll. Physicians Surg. Pak. JCPSP.

[34] Al Abbas D.M., Aman F.S., Almuhaimeed R.S., Almadeh Z.M., Al Abbas D.M., Almadeh Z. (2024). Ruptured Ectopic Pregnancy in a Non-communicating Rudimentary Horn at 18 Weeks of Gestation. Cureus.

[35] Keikha F., Shbeeb H., Homam H., Nasiri Khormoji N., Nouri M. (2024). Cornual Pregnancy as a Rare Entity of Ectopic Pregnancy After Assisted Reproductive Therapy-Embryo Transfer (ART-ET): A Case Report. Clin. Case Rep..

[36] Strawbridge L., Crouch N., Cutner A., Creighton S. (2007). Obstructive Mullerian anomalies and modern laparoscopic management. J. Pediatr. Adolesc. Gynecol..

[37] Ludwin A., Ludwin I., Kudla M., Kottner J. (2015). Reliability of the European Society of Human Reproduction and Embryology/European Society for Gynaecological Endoscopy and American Society for Reproductive Medicine classification systems for congenital uterine anomalies detected using three-dimensional ultrasonography. Fertil. Steril..

[38] Ludwin A., Neto M.A.C., Ludwin I., Nastri C.O., Costa W., Acién M., Alcazar J.L., Benacerraf B., Condous G., DeCherney A. (2020). Congenital Uterine Malformation by Experts (CUME): Diagnostic criteria for T-shaped uterus. Ultrasound Obstet. Gynecol..

[39] Violette C.J., Mandelbaum R.S., Doody K.J., Guner J.Z., Quinn M.M., Ho J.R., Matsuo K. (2022). Obstetric outcomes in women with congenital uterine anomalies: A big data approach. Fertil. Steril..

[40] Yoshihara T., Okuda Y., Yoshino O. (2025). Diagnosis of arcuate uterus using three-dimensional transvaginal ultrasound and investigation of its association with perinatal complications. Int. J. Gynecol. Obstet..

[41] Vaz S.A., Dotters-Katz S.K., Kuller J.A. (2017). Diagnosis and management of congenital uterine anomalies in pregnancy. Obstet. Gynecol. Surv..

[42] Abe J., Nasu T., Noro A., Tsubaki J. (2024). An unusual case of severe asphyxia with the fetal position unexpectedly inverted in a malformed uterus: A case report. J. Med. Case Rep..

[43] Gandelsman E., Grin L., Wainstock T., Berkovitz Shperling R., Scherbina E., Saar-Ryss B. (2025). Risk of adverse pregnancy outcomes after abnormal hysterosalpingography. Hum. Fertil..

[44] Yoshihara T., Okuda Y., Yoshino O. (2024). Association of congenital uterine anomaly with abnormal placental cord insertion and adverse pregnancy complications: A retrospective cohort study. J. Matern. Fetal Neonatal Med..

[45] Cahen-Peretz A., Sheiner E., Friger M., Walfisch A. (2019). The association between Müllerian anomalies and perinatal outcome. J. Matern. Fetal Neonatal Med..

[46] Silsby Z.O., Rhodes S., Kaelber D.C., Sheyn D., Lappen J.R. (2024). 325 Adverse pregnancy outcomes in patients with congenital uterine anomalies: Evaluation of a large population database. Am. J. Obstet. Gynecol..

[47] Akkuş Z.C., Celik O.Y., Karadeniz R.S. (2024). How Often Do We Discover an Abnormality of The Uterus at Delivery? Single Center Experience. Türk Kadın Sağlığı Ve Neonatoloji Derg..

[48] Abhinaya M., Rani B.U., Adilakshmi V., Babu N.B. (2024). A Study of Mullerian Anomalies in Pregnancy: Case Series in a Tertiary Care Centre. Int. J. Med. Public Health.

[49] Gaily A.S., Abdulaal N.A., Alzahrani A., Gaily A., Abdulaal N.A. (2024). A Full-Term Pregnancy in a Patient with Uterus Didelphys. Cureus.

[50] Moraru L., Mitranovici M.-I., Chiorean D.M., Moraru R., Caravia L., Tiron A.T., Cotoi O.S. (2023). Adenomyosis and Its Possible Malignancy: A Review of the Literature. Diagnostics.

[51] Etrusco A., Buzzaccarini G., Laganà A.S., Chiantera V., Vitale S.G., Angioni S., D’alterio M.N., Nappi L., Sorrentino F., Vitagliano A. (2024). Use of diode laser in hysteroscopy for the management of intrauterine pathology: A systematic review. Diagnostics.

